# The effects of tricyclic antidepressants on breast cancer risk

**DOI:** 10.1038/sj.bjc.6600013

**Published:** 2002-01-07

**Authors:** C R Sharpe, J-P Collet, E Belzile, J A Hanley, J-F Boivin

**Affiliations:** Centre for Clinical Epidemiology and Community Studies, Sir Mortimer B. Davis-Jewish General Hospital, 3755 Chemin de la Côte Ste Catherine, Montréal, Québec, Canada H3T 1E2; Department of Medicine, McGill University, Montréal, Québec, Canada H3G 1Y6; Joint Departments of Epidemiology and Biostatistics and of Occupational Health, McGill University, 1020 Pine Ave. West, Montréal, Québec, Canada H3A 1A2

**Keywords:** breast neoplasms, antidepressive agents, epidemiology, case–control studies

## Abstract

To test the hypothesis that tricyclic antidepressant use increases invasive female breast cancer incidence, we carried out a case–control study within the population of female beneficiaries of the Saskatchewan Prescription Drug Plan aged ⩾35 years from 1981–1995 with no history of cancer since 1970. This agency has provided full or partial coverage for outpatient prescriptions to Saskatchewan residents since 1975. We accrued 5882 histologically proven cases and 23 517 controls, randomly selected from the source population and individually matched on age and sampling time. Heavy exposure to any tricyclic antidepressants was associated with an elevated rate ratio for breast cancer 11–15 years later (2.02, 95% confidence interval: 1.34–3.04). *Post hoc* analyses based on the results of genotoxicity studies carried out using *Drosophila melanogaster* suggested that the increased risk could be attributed to the use of the six genotoxic tricyclic antidepressants, and not to the use of the four nongenotoxic tricyclic antidepressants. However, our results may have been confounded by the effects of other determinants of breast cancer associated with tricyclic antidepressant use.

*British Journal of Cancer* (2002) **86**, 92–97. DOI: 10.1038/sj/bjc/6600013 www.bjcancer.com

© 2002 The Cancer Research Campaign

## 

Concerns about the safety of tricyclic antidepressants (TCAs) (Marx, 1992; Miller, 1993; Steingart and Cotterchio, 1995), brought about by the results of animal experiments designed to identify potential carcinogens, raise the issue of whether TCAs are involved in the development of breast cancer.

To study the effects of TCA use on breast cancer risk, we carried out a population-based case–control study, using data obtained from the records of the Saskatchewan Cancer Agency (Canada) and the Saskatchewan Prescription Drug Plan, which has provided outpatient drug coverage to the Saskatchewan population since 1975.

## SUBJECTS AND METHODS

### Study populations

The source population consisted of the women aged ⩾35 years, eligible to benefit from the Saskatchewan Prescription Drug Plan during 1981 to mid-1995, with no history of cancer since 1970 other than non-melanoma skin cancer or carcinoma *in situ* of the cervix. From this population we selected cases of histologically proven invasive female breast cancer and controls (4/case), matched on age and sampling date. Further details can be found elsewhere (Sharpe *et al*, 2000).

To be in our study, subjects must have been eligible to benefit from the Saskatchewan Prescription Drug Plan for ⩾five years before the date of diagnosis of the cases or the sampling date of the controls; hereafter, both dates will be designated as the ‘index date’. This ensured that sufficiently long records of their drug use, if any, would be available for analysis.

### Databases and linkage methods

Information in the databases of the Saskatchewan Prescription Drug Plan and the Saskatchewan Cancer Agency was linked electronically using subjects' unique personal health care identification numbers; see Rawson *et al* (1992).

### Drug exposure data

Exposure data were obtained from the Saskatchewan Prescription Drug Plan database for the period between the index data and January 1, 1976 or the date upon which a subject first became eligible to benefit from the Saskatchewan Prescription Drug Plan, whichever was later. The records of exposure, if any, ranged from 5–19.5 years in length. Because of the small numbers with information available for years 16–19.5, we did not use the information collected then.

The following data were extracted for each antidepressant outpatient prescription: the dispensing date, the class and drug identity according to the American Hospital Formulary System, the number of pills dispensed, and the strength (mg/pill). The daily dose and treatment duration pertaining to each prescription were not available.

No data were available from 1 July 1987 to December 31, 1988, because at that time the Saskatchewan Prescription Drug Plan did not record most drug dispensing to individuals (Rawson *et al*, 1992). The database also lacked information on drugs dispensed in hospitals and as samples, as well as drugs dispensed before being listed in the Saskatchewan Formulary and drugs covered under the Exception Drug Program, unless the physician applied for coverage and it was approved.

### Ethical issues and confidentiality

Extraction of data from the electronic databases of the Saskatchewan Cancer Agency and the Saskatchewan Prescription Drug Plan was carried out by employees of the Saskatchewan Cancer Agency and Saskatchewan Health. The data delivered to the investigators contained no identifying information. The study was approved by the Internal Review Board of the Saskatchewan Cancer Agency, the Cross Agency Study Committee of Saskatchewan Health, and the Sir Mortimer B Davis-Jewish General Hospital Ethics Committee.

### Statistical analysis

We analyzed exposure to TCAs either as a single class of drugs or as two separate classes based on their chemical structures.

To study the effects of exposure timing we *a priori* divided time preceding the index date into five periods: months 1–6, months 7–12, years 2–5, years 6–10, and years 11–15. Exposure during each was characterized with the average rate of dispensing TCAs, which was based on the average number of moles of each different TCA dispensed during each period (m_i_=average moles day^−1^ dispensed for TCA_i_ during a given period). The sum of the averages, i.e. Σm_i_ for all the TCAs dispensed during a given period, represented the measure of exposure. The number of moles dispensed for each drug was calculated from the molecular weight given in the Merck Index (Budavari *et al*, 1989: see Table 1

**Table 1:**
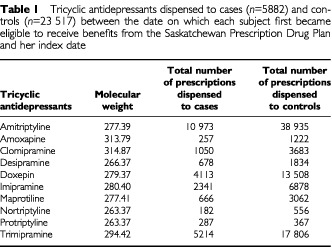
Tricyclic antidepressants dispensed to cases (*n*=5882) and controls (*n*=23 517) between the date on which each subject first became eligible to receive benefits from the Saskatchewan Prescription Drug Plan and her index date

).

We estimated the duration of drug use during the different periods of time by dividing them into 3-month intervals (91 days) and counting the number during which a prescription was dispensed; e.g., drug use during 11 of 20 intervals was 55% of the time. We could not estimate the overall duration of use, because no drug dispensing data were available prior to 1976, from July 1, 1987 to December 31, 1988, and for immigrants to Saskatchewan prior to their becoming eligible to receive benefits from the Prescription Drug Plan.

If the drug exposure history for a period was missing or incomplete due to the reasons listed above, the subject was assigned to a separate exposure category designated ‘other’ (Huberman and Langholz, 1999).

Details of the statistical methods used can be found elsewhere (Sharpe *et al*, 2000). We followed Miettinen's view that exposures during ‘*different time periods represent separate determinants* … *mutually confounded*, and thus requiring joint representation in the same model’ (Miettinen, 1985).

## RESULTS

We accrued 5882 cases and 23 517 controls. The mean age of the cases at diagnosis was 64.1 years (13.3 s.d.). Some 18.7% of the cases and 18.6% of the controls had received a prescription for a TCA.

The ‘crude’ and ‘adjusted’ analyses in Table 2

**Table 2:**
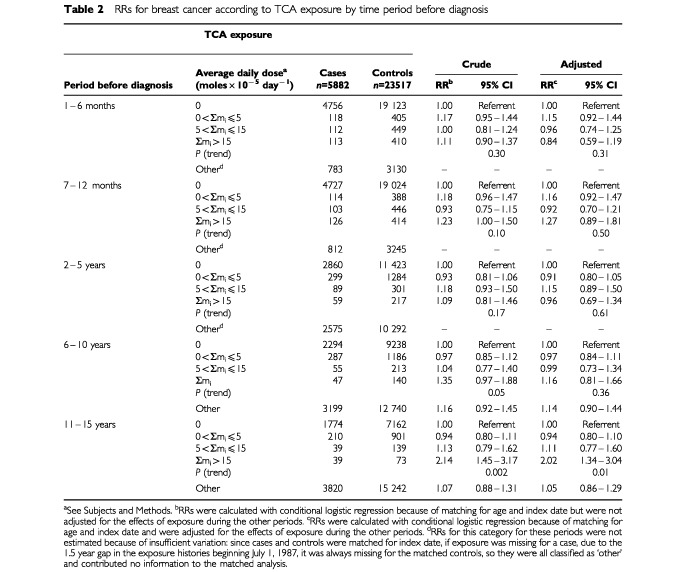
RRs for breast cancer according to TCA exposure by time period before diagnosis

show the rate ratios (RRs) for breast cancer associated with any TCA exposure during each period without and with control for any TCA exposure during the other periods, respectively. The adjusted model shows that only TCA exposure during years 11–15 was associated with a significantly increased risk of breast cancer: among the highly exposed (Σm_i_>15 moles×10^−5^ day^−1^) the incidence was twice that of the unexposed (RR=2.02, 95% CI: 1.34–3.04).

Similar analyses were carried out with exposure expressed as the duration of any TCA use during each period. The overall pattern of results was similar. For TCA use 71–100% of the time during years 11–15, the adjusted RR was 1.52 (95% CI: 1.03–2.26).

The >10 year delay between TCA exposure and the increase in the RR for breast cancer suggested to us that the drugs might be acting as tumour initiators rather than as tumour promoters. Therefore, we searched the literature for evidence of genotoxicity associated with TCA use.

Van Schaik and Graf (1991, 1993) evaluated TCAs using a genotoxicity assay involving wing development in *Drosophila melanogaster* aimed at identifying potential carcinogens. They found that amitriptyline, maprotiline, nortriptyline, and protriptyline were not genotoxic, whereas clomipramine, desipramine, and imipramine clearly were. Since the former four compounds have a carbon atom at position five in the six- or seven-membered central ring, and the latter three have a nitrogen at that position in the seven-membered central ring, they hypothesized that the nitrogen atom in the seven-membered central ring was responsible for the genotoxicity. Recently, Graf (personal communication) evaluated the genotoxicity of amoxapine, doxepin, and trimipramine using the same assay. As expected on the basis of the structural hypothesis, trimipramine was genotoxic. Although doxepin has a carbon at position five, its structure is atypical in that an oxygen atom occupies position eleven in the central ring (Budavari *et al*, 1989); it was also genotoxic. Several aspects of the structure of amoxapine are atypical (Budavari *et al*, 1989); the central ring includes both a nitrogen and an oxygen atom – it too was genotoxic.

Accordingly, we decided to test the hypothesis that the use of genotoxic TCAs was associated with an increased risk of breast cancer.

We defined exposure in terms of Σm_i_ for the two classes of TCAs, based on their genotoxicity in *Drosophila*: the nongenotoxic TCAs (amitriptyline, maprotiline, nortriptyline, and protriptyline), and the genotoxic TCAs (amoxapine, clomipramine, desipramine, doxepin, imipramine, and trimipramine). Since some subjects used drugs from both classes, we included variables representing exposure to each class in a single logistic model to control possible confounding of the effects of one drug class by the other. Table 3

**Table 3:**
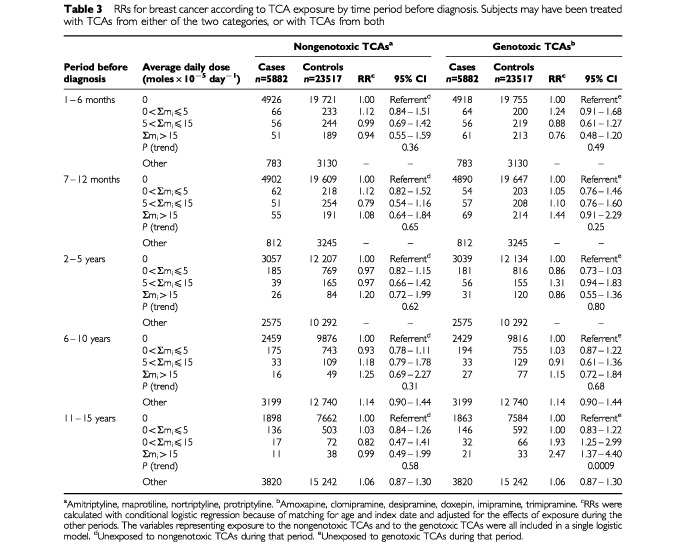
RRs for breast cancer according to TCA exposure by time period before diagnosis. Subjects may have been treated with TCAs from either of the two categories, or with TCAs from both

(left panel) shows that exposure to the nongenotoxic TCAs was not associated with any increased risk. However, increasing exposure to the genotoxic TCAs was associated with a trend towards an increasing RR 11–15 years later (*P*-trend=0.0009; right panel, Table 3). At the highest exposure level the RR was 2.47 (95% CI: 1.38–4.40).

We also carried out an analysis in which potential confounding between the two drug classes was controlled by restriction. Exposure was defined during each period using mutually exclusive categories: exclusive use of TCAs from each class, as well as exposure to both classes. Since there was a common referent for each period consisting of subjects with no exposure to any TCA, the ratio of the RRs associated with exclusive exposure to each class provided an estimate of the ratio of the incidence of breast cancer among subjects exposed to one class of TCAs to the incidence among subjects exposed to the other. At the highest exposure level the incidence associated with exclusive exposure to the genotoxic TCAs during years 11–15 was 2.3 times greater than the incidence associated with exclusive exposure to the nongenotoxic TCAs. The RRs used in this calculation were 1.92 (95% CI: 0.93–3.95) and 0.84 (95% CI: 0.36–1.93), respectively.

We also carried out an analysis like that of Table 3 in which exposure was expressed as the estimated duration of use during each period; see Table 4

**Table 4:**
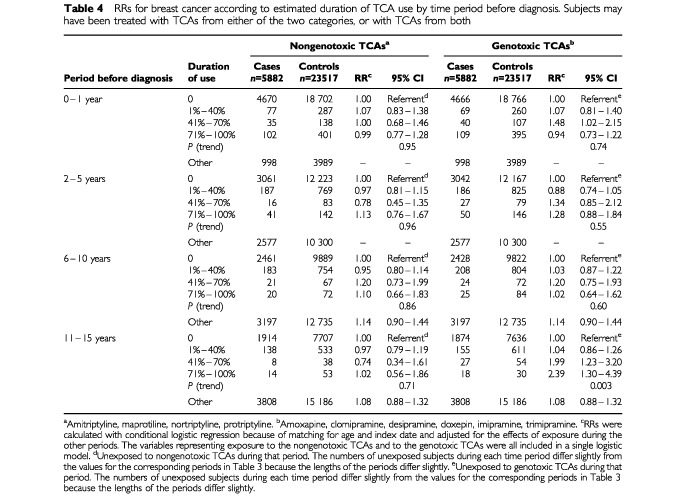
RRs for breast cancer according to estimated duration of TCA use by time period before diagnosis. Subjects may have been treated with TCAs from either of the two categories, or with TCAs from both

. There was no increased risk associated with use of the nongenotoxic TCAs, but for the genotoxic TCAs there was a trend towards an increasing RR with increasing duration of use during years 11–15 (*P*-trend=0.003). For drug use 71–100% of the time during that period the RR was 2.39 (95% CI: 1.30–4.39). The corresponding analysis using restriction to control confounding showed that at the highest exposure level the incidence associated with exclusive exposure to the genotoxic TCAs during years 11–15 was 2.4 times greater than the incidence associated with exclusive exposure to the nongenotoxic TCAs. The RRs used in this calculation were 1.90 (95% CI: 0.93–3.90) and 0.80 (95% CI: 0.40–1.61), respectively.

## DISCUSSION

We found that increasing exposure to any TCAs during years 11–15 was associated with a trend towards an increasing RR for breast cancer (Table 2). *Post hoc* analyses based on classifying the drugs according to their genotoxicity in *Drosophila* suggest that the use of genotoxic TCAs was responsible for the increased risk (amoxapine, clomipramine, desipramine, doxepin, imipramine, trimipramine), while the use of the nongenotoxic TCAs was not associated with an increased risk (amitriptyline, maprotiline, nortriptyline, protriptyline).

Because our accrual of cases from the source population was nearly complete (Parkin *et al*, 1997) and our exposure data were collected routinely before the index date, there was little potential for selection and recall bias concerning exposure.

Since our measures of exposure were based on outpatient prescriptions, actual consumption of TCAs probably differed from our estimates. It is unlikely that all drugs dispensed were ingested. If we overestimated exposure, then the slope of the dose-risk relationship that we observed would be less than the true slope (MacMahon and Trichopoulos, 1996). Although we may have underestimated exposure, since we had no information about some of the TCAs dispensed (see Subjects and Methods), these amounts were probably small relative to the amounts used in calculating exposure.

Could our results be confounded by the effects of other determinants of breast cancer associated with TCA use?

Although depression was once considered to be associated with the development of cancer as the result of immunologic and endocrine dysfunction, there is now little supporting evidence (Linkins and Comstock, 1990; Spiegel, 1996). Although Penninx *et al* (1998) recently found that the incidence of cancer was 1.9 times greater (95% CI: 1.1–3.1) among patients with chronic depression, they obtained information about antidepressant use for only the 2 weeks period preceding inception of the cohort. Although they considered the possibility that antidepressant drug use may have increased the risk of cancer and controlled for it, it is unlikely that they controlled adequately for antidepressant drug use with such limited information. Furthermore, they did not consider that some antidepressants might be more hazardous than others. Similarly, Gallo *et al* (2000) obtained information on depression status at baseline and cancer at follow-up 13 years later for 2017 persons. They found no overall association between depression and the development of cancer. Among women, however, there was an association between major depression and breast cancer. The RR adjusted for age, tobacco use, and alcohol abuse was 3.8 (95% CI: 1.0–14.3); this estimate was based on 25 breast cancer cases. They reported that adjustment ‘for use of psychotropic medicines and use of health-care services at baseline’ did not change this estimate substantially. No further details of this adjustment were provided, nor were they able to study the interval between major depression and the diagnosis of cancer.

Nevertheless, if other determinants of breast cancer (Sharpe and Boivin, 2000) were associated with TCA use, it is possible that our results might be attributable to confounding by such determinants. It is unlikely, however, that strong positive confounding associated with the use of the genotoxic TCAs led to an apparent effect stronger than the overall effect of TCA exposure (compare the right panels of Tables 3 and 4 to the right panel of Table 2), while strong negative confounding associated with the use of the nongenotoxic TCAs led to an apparent lack of effect. TCAs are usually prescribed for depression without consideration of subtle details of their chemical structures and without consideration of breast cancer as an adverse effect. Exposure to the genotoxic TCAs may have increased the risk of breast cancer, while exposure to the nongenotoxic TCAs may have had no effect on risk.

Since selection bias, recall bias, exposure misclassification, and chance are unlikely explanations for our findings, and differential patterns of confounding according to the chemical structures of the drugs seem unlikely, a biologic explanation may be required. The >10 year delay between exposure to the genotoxic TCAs and the increase in the RR for breast cancer is suggestive of tumour initiation rather than tumour promotion. Ionizing radiation, the prototypical initiator, increases the risk of breast cancer after a delay of 10 years (Land, 1987).

The results of TCA genotoxicity assays involving bacteria, *Drosophila*, and human lymphocytes are conflicting (van Schaik and Graf, 1991). The consistency between the results of the assays carried out by van Schaik and Graf (1991, 1993) and Graf (2001) in *Drosophila* and our epidemiologic findings suggests that it may be the most appropriate.

Although several epidemiologic studies found no positive associations between TCA use and the development of cancer (Danielson *et al*, 1982; Friedman and Ury, 1980, 1983; Kelly *et al*, 1999; Selby *et al*, 1989; Weiss *et al*, 1998), they were limited either by small sample size, self-reporting of use, failure to specify dosage, duration of use, or timing of use, and lack of control of confounding. Recently, Wang *et al* (2001) followed 38 273 women who filled a prescription for an antidepressant over a period of up to 24 months, and 32 949 women who filled a prescription for any other medication over the same period. Both groups were followed for a maximum of 7.5 years. TCA use was not associated with the development of breast cancer (RR=1.09; 95% CI: 0.92–1.31), which is consistent with our finding that TCA use was not associated with any increase in risk with until at least 10 years had elapsed.

Two other studies have reported positive associations between TCA use and the development of breast cancer. Wallace *et al* (1982) carried out a case–control study of antidepressant use (TCAs or phenelzine) in relation to breast cancer incidence and obtained an adjusted RR=2.8 (*P*<0.04) for use >1 month. In a similar study, Cotterchio *et al* (2000) found that TCA use for ⩾25 months was associated with an adjusted RR=2.1 (95% CI: 0.9–5.0). Neither study specified dosage or the timing of use.

Another two studies reported positive associations between antidepressant use and the development of other cancers. Harlow and Cramer (1995) carried out a case–control study of ovarian cancer incidence and obtained an adjusted RR=2.1 (95% CI: 0.9–4.8) for any prior use of antidepressants or benzodiazepines lasting ⩾6 months. Among women who first used these drugs before age 50 years the RR was 3.5 (95% CI: 1.3–9.2). Among those who used them ⩾10 years before diagnosis, the RR was 9.7 (95% CI: 1.2–78.8). These findings, however, were not corroborated by Coogan *et al* (2000). Both studies were based on self-reported exposures. Dalton *et al* (2000) conducted a population-based cohort study in Denmark using the nation's prescription database. They followed 30 807 antidepressant users aged ⩾15 years for up to 7 years (mean=3.2 years) and found an increased risk of non-Hodgkin's lymphoma among subjects who received ⩾5 prescriptions for TCAs (standardized incidence ratio=2.5; 95% CI: 1.4–4.2).

## CONCLUSION

Taken together, our results and those of others suggest that the relations between TCA use and the development of breast cancer and other cancers as well should be evaluated further in studies designed specifically to test the hypothesis that the TCAs found to be genotoxic by van Schaik and Graf (1991, 1993) and Graf (personal communication), are carcinogenic, in which potential confounding by other determinants can be controlled.
